# Accurate treatment effect estimation using inverse probability of treatment weighting with deep learning

**DOI:** 10.1093/jamiaopen/ooaf032

**Published:** 2025-04-26

**Authors:** Junghwan Lee, Simin Ma, Nicoleta Serban, Shihao Yang

**Affiliations:** H. Milton Stewart School of Industrial and Systems Engineering, Georgia Institute of Technology, Atlanta, GA 30332, United States; H. Milton Stewart School of Industrial and Systems Engineering, Georgia Institute of Technology, Atlanta, GA 30332, United States; H. Milton Stewart School of Industrial and Systems Engineering, Georgia Institute of Technology, Atlanta, GA 30332, United States; H. Milton Stewart School of Industrial and Systems Engineering, Georgia Institute of Technology, Atlanta, GA 30332, United States

**Keywords:** treatment effect estimation, propensity score, electronic health records, deep learning

## Abstract

**Objectives:**

Observational data have been actively used to estimate treatment effect, driven by the growing availability of electronic health records (EHRs). However, EHRs typically consist of longitudinal records, often introducing time-dependent confounding that hinder the unbiased estimation of treatment effect. Inverse probability of treatment weighting (IPTW) is a widely used propensity score method since it provides unbiased treatment effect estimation and its derivation is straightforward. In this study, we aim to utilize IPTW to estimate treatment effect in the presence of time-dependent confounding using claims records.

**Materials and Methods:**

Previous studies have utilized propensity score methods with features derived from claims records through feature processing, which generally requires domain knowledge and additional resources to extract information to accurately estimate propensity scores. Deep learning, particularly using deep sequence models such as recurrent neural networks and Transformer, has demonstrated good performance in modeling EHRs for various downstream tasks. We propose that these deep sequence models can provide accurate IPTW estimation of treatment effect by directly estimating the propensity scores from claims records without the need for feature processing.

**Results:**

Comprehensive evaluations on synthetic and semi-synthetic datasets demonstrate that IPTW treatment effect estimation using deep sequence models consistently outperforms baseline approaches, including logistic regression and multilayer perceptrons, combined with feature processing.

**Discussion:**

Our findings demonstrate that deep sequence models consistently outperform traditional approaches in estimating treatment effects, particularly under time-dependent confounding. Moreover, Transformer-based models offer interpretability by assigning higher attention weights to relevant confounders, even when prior domain knowledge is limited.

**Conclusion:**

Deep sequence models enable accurate treatment effect estimation through IPTW without the need for feature processing.

## Introduction

Randomized controlled trials (RCTs) are considered the gold standard to estimate treatment effect.^1^^,^^2^ However, RCTs are not always feasible due to high experimental costs, time constraints, and ethical considerations.^3^^,^^4^ Because observational data are less constrained by the limitations inherent in RCTs, they have become more commonly used in studies aiming to estimate treatment effect.^5^ Electronic health records (EHRs) are an important type of observational data that contain rich information about the medical history of an individual.^6^ With the increasing availability of EHRs in the healthcare domain,^7^ EHRs with different types of data modalities, such as time series physiological measurements, claims records, and clinical notes, have also been used for treatment effect estimation.^4^^,^^8^ While utilizing EHRs can mitigate the limitations of RCTs, EHRs often contain confounding that hinder the unbiased estimation of treatment effect.

*Confounding variables*, also known as *confounders*, are variables that influence both treatment assignment and outcome.^9^ Confounding is present in a study when it includes such variables. EHRs usually contain medical records arranged in chronological order, leading to *time-dependent confounding*. Adjusting for confounding is essential to accurately estimate treatment effect, but it can be particularly challenging with time-dependent confounding since time-dependent confounding depends on the temporal pattern and dependencies of the confounding variables that change over time and are affected by past features.

Propensity score methods are commonly used to adjust for confounding in treatment effect estimation. These methods leverage the balancing property of the propensity scores, which ensures that the distribution of observed covariates is identical between treated and untreated groups when conditioned on the propensity score.^10^ Inverse probability of treatment weighting (IPTW) is a propensity score method that creates a synthetic sample where treatment assignment is independent of the observed covariates by weighting the sample with the inverse of its propensity score. IPTW is widely used since it provides unbiased treatment effect estimation and its derivation is straightforward.^11^^,^^12^

Claims records are a subset of EHRs, containing longitudinal records of standardized codes for diagnoses, medications, and medical procedures. They are a crucial data source for research in medicine and healthcare domains.^8^ While claims records are often used to estimate treatment effect with adjustments for confounding using propensity score methods,^13^^,^^14^ there is a lack of studies focusing on estimating treatment effect using claims records in the presence of time-dependent confounding.

**Figure 1.**
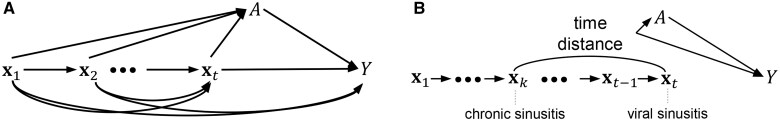
(A) Causal diagram of our problem setup. A denotes binary treatment and Y denotes continuous outcome. A claims record $x_{t}$ includes medical codes and also can include a treatment assignment A. (B) Causal diagram of a hypothetical confounding scenario in our experiment using a semi-synthetic dataset. The confounding depends on the record-wise distance between *chronic sinusitis* and *viral sinusitis*. Arrows between records are omitted for readability.

In this study, we aim to utilize IPTW to estimate treatment effect in the presence of time-dependent confounding using claims records. IPTW can provide an unbiased estimation of treatment effect, but it requires an accurate estimation of the propensity scores to ensure this unbiasedness. The challenge lies in accurately estimating the propensity scores in the presence of time-dependent confounding, as it involves capturing the temporal patterns and dependencies of confounding variables across longitudinal records. For example, Figure 1B depicts our hypothetical scenario of time-dependent confounding in the experiment using a semi-synthetic dataset, where the confounding depends on the record-wise distance between the code of chronic sinusitis and viral sinusitis. Previous studies have utilized propensity score methods with features derived from claims records through feature processing. However, such feature processing usually requires domain knowledge and additional resources to extract information to accurately estimate propensity score.^15^

Deep learning, particularly using deep sequence models such as recurrent neural networks (RNNs) and Transformer, has demonstrated impressive performance in modeling sequential data^16^^,^^17^ and has been successfully adopted for modeling EHRs in various tasks.^18–20^ We propose that deep sequence models based on these architectures can provide accurate IPTW estimation of treatment effect by directly estimating the propensity scores from claims records, even in the presence of time-dependent confounding, without the need for feature derivation from the EHRs. We empirically demonstrate this through comprehensive evaluations, comparing baseline methods that employ feature processing with deep sequence models, using synthetic and semi-synthetic datasets. For the baseline methods, we select logistic regression and multi-layer perceptions, the 2 most widely used methods for propensity score estimation with feature processing. Our results show that IPTW with deep sequence models provides better estimates of treatment effect compared to baseline methods, without requiring additional feature processing.

## Methods

### Preliminaries

#### Potential outcome framework and average treatment effect

Consider a binary treatment setting with 2 possible treatments: treated (A=1) and control (A=0). The potential outcome framework assumes each individual i has a pair of potential outcomes $Y_{A=1}^{\left(i\right)}$ and $Y_{A=0}^{\left(i\right)}$. For notational simplicity, we denote these potential outcomes as $Y_{1}^{\left(i\right)}$ and $Y_{0}^{\left(i\right)}$, respectively. The average treatment effect (ATE) is defined as follows:


#(1) E[Y1-Y0]=E[Y1]-E[Y0].

However, ATE cannot be directly calculated from eqn (1) since only one of the potential outcomes, either $Y_{1}$ or $Y_{0}$, can be observed for each individual.^21^^,^^22^ When treatment assignment is random, we know that $E[Y|A=1]=E[Y_{1}]$ and $E[Y|A=0]=E[Y_{0}]$. Therefore, under randomized treatment assignment, ATE can be calculated as


#(2) E[Y1-Y0]=E[Y|A=1]-E[Y|A=0].

The ATE calculation does not generally hold in observational studies where randomization of treatment assignment cannot be guaranteed and confounding exists.

#### Propensity score

Propensity score, which indicates the probability of an individual being assigned to treatment given the observed covariates of the individual,^10^ is defined as follows:


#(3) e(x)=P(A=1|x),

where x is a covariate vector. In observational studies, the true propensity score is typically unknown and needs to be estimated:


#(4) e^=fθ(x),

where $f_{\theta }$ is a function with a parameter set θ that maps x to the estimated propensity score $\hat{e}$.

#### ATE estimation using inverse probability of treatment weighting

In this study, we use IPTW to estimate the ATE. The ATE can be estimated using IPTW as follows:

#(5) ΔIPTW^=1N(∑i=1NA(i)Y(i)e(i)^-∑i=1N(1-A(i))Y(i)(1-e(i)^)),

where N is the total number of samples in the dataset and $\hat{e^{\left(i\right)}}$ is the estimated propensity score of individual i. In eqn (5), IPTW constructs a synthetic population by up-weighting treated samples with low propensity scores (ie, samples unlikely to receive the treatment) and untreated samples with high propensity scores (ie, samples likely to receive the treatment). At the same time, it down-weights treated samples with high propensity scores (ie, samples likely to receive the treatment) and untreated samples with low propensity scores (ie, samples unlikely to receive the treatment). This reweighting creates a balanced synthetic population where treatment assignment is independent of the covariates. Based on the potential outcome framework, $\hat{\Delta_{\text{IPTW}}}$ in eqn (5) is an unbiased estimation of the ATE.^11^^,^^23^

Compared to propensity score matching, which is another widely used propensity score method, IPTW offers a more straightforward implementation through eqn (5). In contrast, propensity score matching requires selecting a matching method (ie, how to pair treated and untreated samples) and addressing issues related to unmatched samples.^24^^,^^25^ Throughout this paper, we refer to the estimated ATE using IPTW, calculated by the eqn (5), as the estimated ATE.

### Problem setup

Figure 1A illustrates the causal diagram of our problem setup. We consider a claims records dataset $D={\left\{\left\{x_{t}^{\left(i\right)}{\}}_{t=1}^{T^{\left(i\right)}}\cup \{A^{\left(i\right)},Y^{\left(i\right)}\right\}\right\}}_{i=1}^{N}$, which consists of records from N independent samples. Each sample i contains records at $T^{\left(i\right)}$ discrete time points. We assume that a binary treatment A ∈ {0,1} and a continuous real-valued outcome Y∈R are observed for each sample i at the observation end time point. We can view each record as a bag of medical codes that can be represented using multi-hot encoding. For instance, a record containing medical codes for *fever* and *acetaminophen* can be expressed as a bag with *fever* and *acetaminophen*. Each record can then be represented as a multi-hot encoded vector, where the elements for these codes are ones, and the rest are zeros. Therefore, for each sample i, we observe a record $x_{t}^{\left(i\right)}\in \{0,1{\}}^{d_{x}}$ at time t, where $d_{x}$ denotes the dimensionality of the records (ie, the number of unique medical codes). For better understanding of the claims records and their multi-hot encoded representations, we provide a few sample records in the Supplementary Material.

Our objective is to estimate the ATE using IPTW as defined in eqn (5) on the claims records dataset D that may contain time-dependent confounding. Specifically, we seek to obtain a model $f_{\theta }$ that maps $X^{\left(i\right)}$ to the estimated propensity score $\hat{e^{\left(i\right)}}$ as

#(6) e(i)^=fθ(X(i)),

where $X^{\left(i\right)}$ is the set of records for sample i. A desirable model would be able to estimate $\hat{e^{\left(i\right)}}$ as closely as possible to the true propensity score $e^{\left(i\right)}$, thereby providing an accurate estimation of the ATE using IPTW.

### Recurrent neural networks

Recurrent neural networks (RNNs) are commonly used deep sequence model architectures with a long history of being used to model sequential data. RNNs have shown significant success in various tasks, such as machine translation^16^ and speech recognition.^26^ Due to the sequential nature of EHRs, RNNs have also been actively used in modeling EHRs for various medical tasks. For example, Lipton et al^27^ used Long Short-Term Memory Networks (LSTM)^28^ and Choi et al^29^ used Gated Recurrent Units (GRU)^30^ to predict medical codes of patients in intensive care units, respectively. Additionally, Lee et al^31^ used GRU to predict the mortality of COVID-19 patients.

**Figure 2.**
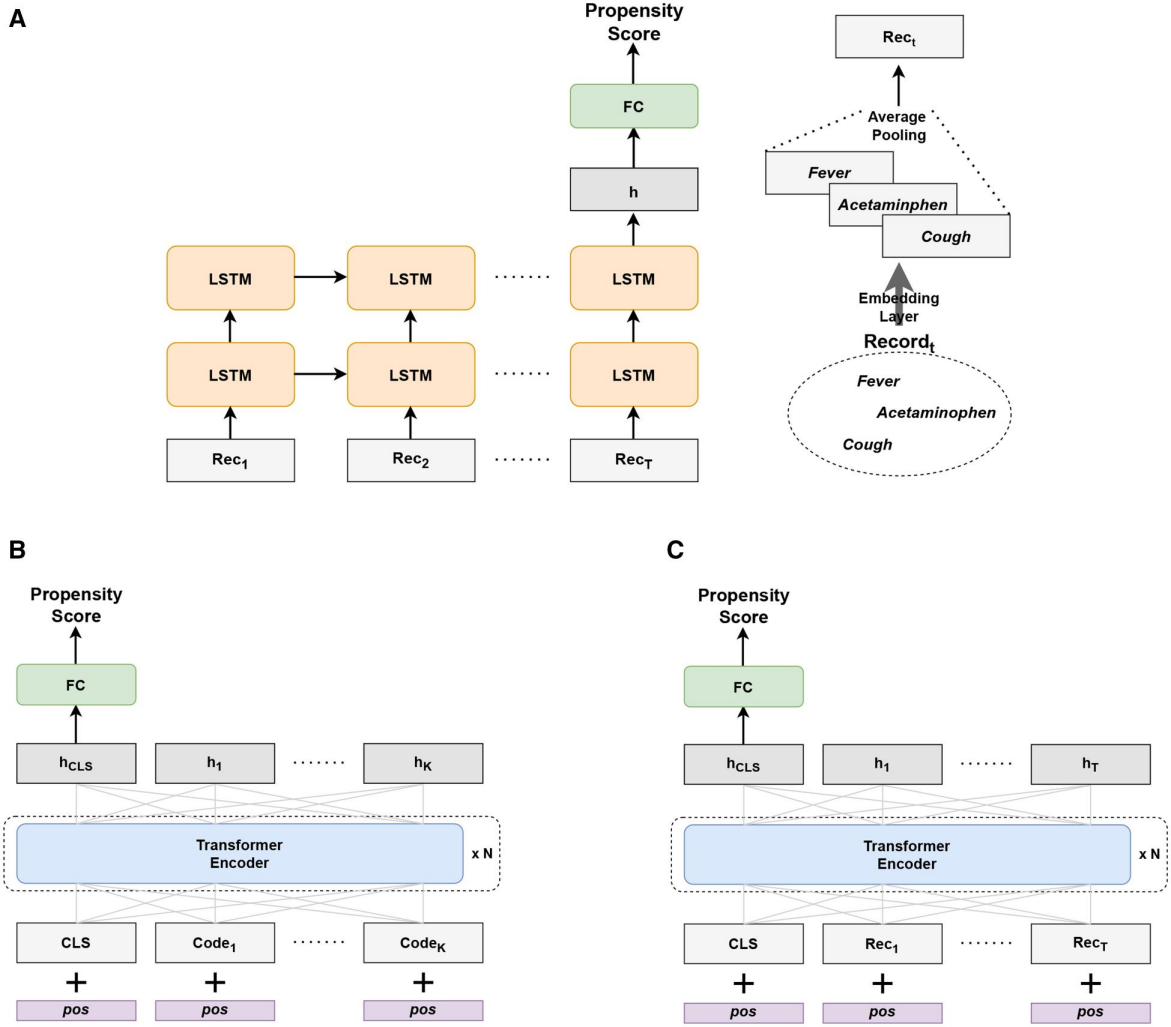
(A) LSTM to estimate propensity score using claims records. Average pooling is applied to the code representations to generate record representation, aggregating the representations of the codes present in the record. For example, if ${\text{Record}}_{t}$ contains *Fever*, *Acetaminophen*, and *Cough* codes, the record representation of ${\text{Record}}_{t}$ is generated by averaging the representations of these 3 codes. (B) ${\text{BERT}}_{\text{code}}$ to estimate propensity score using claims records. The input representations are constructed by arranging code representations in chronological order. (C) ${\text{BERT}}_{\text{record}}$ to estimate propensity score using claims records. The input representations are constructed using record representations, similar to LSTM.

We employ LSTM as the model $f_{\theta }$ to estimate the propensity scores in accordance with our problem setup. Figure 2A visually illustrates how LSTM estimates the propensity scores using claims records. Initially, we apply average pooling to obtain a representation for each record, which aggregates the representations of the codes present in the record (ie, codes in the bag of records). Code representations are generated using an embedding layer. The record representations are then sequentially fed into the LSTM. A fully connected neural network layer with sigmoid activation is applied to the final hidden state output by the LSTM to estimate the propensity scores. The model is trained by minimizing the cross-entropy loss between the estimated propensity scores and the binary treatment assignment.

### Transformer

Transformer,^17^ originally developed for machine translation, has demonstrated exceptional performance and has since become the foundation for a wide range of natural language processing tasks. Designed with an encoder-decoder structure, the original Transformer comprises multiple identical multi-head self-attention layers in both encoder and decoder. Building on this architecture, encoder-only and decoder-only models were introduced by stacking Transformer encoder and decoder layers, respectively. Notably, BERT (Bidirectional Encoder Representations from Transformers)^32^ and GPT (Generative Pre-Training)^33^ are pioneering models based on encoder-only and decoder-only architectures, respectively. In particular, BERT has been extensively utilized for modeling EHRs, adopting transfer learning paradigms similar to those in NLP. This approach involves pre-training the model on large-scale EHR datasets, followed by fine-tuning for specific downstream medical tasks.^34–37^

We use BERT as the model $f_{\theta }$ to estimate propensity scores using claims records based on our problem setup. Specifically, we use 2 different schemes to construct input representations for BERT. In the first scheme, we construct an input representation by organizing code representations in chronological order. The code representations are generated using an embedding layer, and positional encodings are added to the code representations to incorporate positional information. The codes from the t-th record are added with the corresponding positional encodings based on their positions. Figure 2B provides an illustration of how BERT is utilized for estimating propensity scores using claims records with the first input representation scheme. This input representation scheme is commonly employed in existing studies that leverage BERT for modeling EHRs.^34^^,^^36^ In the second scheme, we apply average pooling to obtain record representations, which is similar to the approach used in LSTM. Subsequently, the record representations are organized chronologically and added with positional encodings based on their positions within the order. Figure 2C visually depicts the use of BERT for estimating propensity scores using claims records with the second input representation scheme.

We refer to BERT with the first input representation scheme as BERT_code_ and BERT with the second input representation scheme as BERT_record_. We use positional encodings based on sine and cosine functions for both schemes, following the original publications.^17^^,^^32^ In both BERT_code_ and BERT_record_, we place [CLS] token at the first position of every input representation and use the learned [CLS] token representation to estimate propensity score. A fully connected neural network layer with sigmoid activation is used to transform the final [CLS] token representation to the estimated propensity score. The model is trained by minimizing the cross-entropy loss between the estimated propensity score and the binary treatment assignment.

## Experiments

We conduct experiments using synthetic and semi-synthetic datasets. Synthetic and semi-synthetic datasets are particularly advantageous since they provide access to ground-truth outcomes for all potential treatments and interventions, which are typically unavailable in real-world datasets.

### Dataset

#### Synthetic dataset

We formulate a synthetic dataset to conduct controlled experiments with potential time-dependent confounding scenarios based on our problem setup. For notational simplicity, we omit the index for each sample. First, we specify the number of samples N in the dataset, the number of records T for each sample, and the dimensionality of the records $d_{x}$. We set N=12 000 and $d_{x}=100$. Each sample is independently generated and contains T total records, where T∼Poisson(λ=10).

Second, we generate static and dynamic variables for each sample. The static variable matrix $B\in R^{T\times d_{x}}$ represents intrinsic characteristics of the sample that influence health status and remain constant over time. Examples of static variables include gender and genetic characteristics. The dynamic variable matrix $C\in R^{T\times d_{x}}$ represents factors that influence health status and can change over time. Examples of dynamic variables include immune system states and socioeconomic status. Both types of variables are independently generated for each sample and are used as parameters to generate the claims records of the sample.

Third, we generate claims records for each sample using the both static and dynamic variables. The occurrence probability of the k-th dimension at time point t is generated from a Beta distribution using the corresponding static and dynamic variables as follows:

$$\;\;\;\;P_{tk}\;∼\;\text{Beta}(B_{tk},C_{tk}),\;\;$$

where $P_{tk}$, $B_{\mathrm{tk}}$, and $C_{tk}$ are the (t,k) entry of occurrence probability matrix $P\in {\left[0,1\right]}^{T\times d_{x}}$, the static variable matrix B, and the dynamic variable matrix C, respectively. Subsequently, the claims record $X\in \{0,1{\}}^{T\times d_{x}}$ is generated by using the Bernoulli distribution as follows:

$$X_{tk}∼\text{Bernoulli}(P_{tk}),$$

where $X_{tk}$ and $P_{tk}$ represent (t,k) entry of the claims record X and occurrence probability matrix P, respectively.

Based on the synthetic dataset generated using the aforementioned process, we design 3 confounding scenarios: *consecutive occurrence*, *occurrence distance*, and *occurrence window*. In the *consecutive occurrence* scenario, higher true propensity scores and outcomes are given to samples with consecutive occurrences of a specific code. The *occurrence distance* scenario allocates higher true propensity scores and outcomes to samples with shorter record-wise distances between the occurrences of 2 specific codes. In the *occurrence window* scenario, samples with more occurrences within a specific lookup window receive higher true propensity scores and outcomes.

Treatment is assigned by using the Bernoulli distribution with the true propensity score of a sample as A∼Bernoulli(e(X)). We assume homogeneous and additive treatment effect for all samples. In order to accurately estimate propensity score under all 3 scenarios, the model needs to learn the temporal patterns of the specific codes that influence the true propensity score. Additional details about the generation of the synthetic dataset and the confounding scenarios are available in the Supplementary Material.

#### Semi-synthetic dataset

We formulate a semi-synthetic dataset using Synthea,^38^ to simulate a potential real-world time-dependent confounding scenario. Synthea is a simulated dataset derived from real-world EHRs.^38^ We consider a hypothetical scenario where we aim to estimate the treatment effect for *viral sinusitis* and modify Synthea to introduce time-dependent confounding as follows: we identify 2 disease codes, *chronic sinusitis* and *viral sinusitis*; and assign higher true propensity scores and outcomes to samples with shorter record-wise distances between occurrences of these 2 codes. We chose *chronic sinusitis* and *viral sinusitis* to create confounding due to their similar symptoms but differing treatments, potentially leading to confounding when estimating the treatment effect of a medication for *viral sinusitis* in the presence of *chronic sinusitis*.^39^ Treatment is assigned using the Bernoulli distribution with the true propensity score of a sample similar to the synthetic data. We also assume homogeneous and additive treatment effect for all samples. Additional details about the semi-synthetic dataset are available in the Supplementary Material.

### Setup

#### Baseline methods

We use logistic regression and multilayer perceptrons (MLP) as baseline methods, which are commonly used for estimating propensity scores. To construct the feature vector for each sample, we sum the occurrences of each code across all records, resulting in a vector where the k-th element represents the occurrence count of the k-th code. Subsequently, we standardize the feature vectors using the mean and standard deviation of occurrences per code calculated from the entire dataset.

In addition, we apply high-dimensional propensity score adjustment (HDPS),^15^ as the feature processing method for the baselines. HDPS is widely used in propensity score methods and adds proxy variables to adjust the confounding in longitudinal EHRs with a large number of variables.^15^ HDPS generates additional recurrence features that capture the recurrence patterns of each code within a sample’s records, then ranks and selects the top k features with the highest potential to control confounding. We implement both the original HDPS setup, as described in the original publication, and the implementation of HDPS without feature selection as the feature processing method for logistic regression and MLP. Implementation details of HDPS is available in the Supplementary Material.

#### Evaluation metrics

We use mean absolute error (MAE) of the propensity score and ATE as evaluation metrics. The MAE of the propensity score is calculated by averaging the absolute errors between the estimated and the true propensity scores on the samples in the evaluation set, which is defined as follows:

#(7) 1M∑i=1M|e(i)-e(i)^|,

where $e^{\left(i\right)}$ and $\hat{e^{\left(i\right)}}$ are the true and estimated propensity score of sample i, respectively, and M is the sample size of the evaluation set. We also use a weighted MAE of the propensity score, which is defined as follows:

#(8) 1M∑i=1Mw(i)|e(i)-e(i)^|,

where $w^{\left(i\right)}$ is the weight corresponding to the error of the estimation for sample i. We use the true propensity score as the weight by setting $w^{\left(i\right)}=e^{\left(i\right)}$, placing more importance to samples with severe confounding in the evaluation.

The MAE of ATE is the absolute error between the estimated and true treatment effect, calculated as $\left|\hat{\Delta_{\text{IPTW}}}-\Delta_{\text{True}}\right|$. Due to the potential instability of the ATE caused by extreme values of the estimated propensity scores,^40^ we also compute the MAE of ATE after applying symmetric trimming and clipping of the estimated propensity score for evaluation with an adjustment of extreme propensity scores. Symmetric trimming^41^ excludes samples from the calculation of the MAE of ATE if their estimated propensity scores fall outside the range [α,1-α], where α is a pre-defined threshold. Symmetric clipping clips the estimated propensity scores with a minimum value of α and a maximum value of (1-α) when computing the MAE of ATE.

#### Implementation details

We evaluate the models and baselines using 10-fold cross-validation on the synthetic dataset and 5-fold cross-validation on the semi-synthetic dataset. Optimal hyperparameters are selected by using a validation set and a subset of training set. Optimal hyperparameters are selected by using a validation set and a subset of training set. We use Adam,^42^ optimizer without learning rate scheduling. Additional details on hyperparameter selection and implementation for the models and baselines can be found in the Supplementary Material.

## Results

The results on the synthetic dataset with 3 confounding scenarios are presented in Table 1. Table 2 shows the results on the semi-synthetic dataset. In both experiments, deep sequence models (LSTM and BERT) outperform the baseline methods in terms of the MAE of the propensity scores and ATE. There were no significant differences observed between the MAE of ATE and MAE of ATE with symmetric trimming and clipping across all models and baselines in both experiments.

**Table 1.** Mean absolute error (MAE) of propensity scores and average treatment effect (ATE) estimates across different methods and baselines on the synthetic dataset with 3 confounding scenarios.

Scenario	Model	PS	PS_W_	ATE	ATE_T_	ATE_C_
*Consecutive occurrence*	LSTM	**0.147 ± .005**	0.076 ± .003	5.52 ± 1.24	5.56 ± 1.30	5.74 ± 1.24
	BERT_code_	0.159 ± .003	**0.074 ± .001**	**3.64 ± 1.75**	**3.64 ± 1.75**	**3.64 ± 1.75**
	BERT_record_	0.162 ± .003	0.083 ± .004	6.71 ± 1.18	6.70 ± 1.18	6.71 ± 1.18
	MLP	0.160 ± .004	0.085 ± .003	7.18 ± .830	7.23 ± .829	7.16 ± .890
	MLP-recurrence	0.186 ± .005	0.097 ± .003	8.53 ± 1.71	8.52 ± 1.75	8.51 ± 1.76
	MLP-HDPS	0.256 ± .003	0.140 ± .002	14.7 ± .677	14.7 ± .660	14.6 ± .662
	LR	0.156 ± .002	0.085 ± .002	7.53 ± 1.13	7.53 ± 1.13	7.53 ± 1.13
	LR-recurrence	0.184 ± .003	0.100 ± .003	9.44 ± .795	9.44 ± .795	9.44 ± .795
	LR-HDPS	0.259 ± .005	0.145 ± .007	16.1 ± 2.13	16.1 ± 2.13	16.1 ± 2.13
*Occurrence distance*	LSTM	**0.079 ± .003**	**0.033 ± .003**	**2.19 ± .983**	**2.14 ± .983**	**2.10 ± 1.00**
	BERT_code_	0.100 ± .007	0.051 ± .002	2.69 ± 1.99	2.70 ± 2.00	2.69 ± 2.00
	BERT_record_	0.113 ± .004	0.055 ± .002	3.69 ± 1.64	3.68 ± 1.63	3.69 ± 1.65
	MLP	0.118 ± .002	0.057 ± .001	4.20 ± 1.20	4.21 ± 1.20	4.20 ± 1.19
	MLP-recurrence	0.123 ± .002	0.058 ± .002	4.65 ± 1.29	4.64 ± 1.33	4.63 ± 1.29
	MLP-HDPS	0.120 ± .002	0.055 ± .002	5.41 ± .969	5.41 ± .969	5.40 ± .970
	LR	0.118 ± .002	0.057 ± .001	4.45 ± .991	4.45 ± .991	4.45 ± .991
	LR-recurrence	0.112 ± .007	0.055 ± .002	3.11 ± 1.79	3.11 ± 1.79	3.11 ± 1.79
	LR-HDPS	0.125 ± .002	0.058 ± .002	5.82 ± 1.15	5.82 ± 1.15	5.82 ± 1.15
*Occurrence window*	LSTM	**0.070 ± .003**	**0.033 ± .001**	3.36 ± 2.16	**2.16 ± 1.45**	**2.27 ± 1.84**
	BERT_code_	0.153 ± .017	0.048 ± .003	**2.40 ± 1.63**	2.45 ± 1.69	2.39 ± 1.61
	BERT_record_	0.112 ± .005	0.057 ± .004	3.39 ± 2.57	3.50 ± 2.61	3.37 ± 2.46
	MLP	0.279 ± .001	0.128 ± .004	10.9 ± 1.01	11.0 ± .989	11.0 ± .444
	MLP-recurrence	0.283 ± .004	0.131 ± .006	11.1 ± 1.13	11.1 ± 1.17	11.1 ± 1.20
	MLP-HDPS	0.304 ± .150	0.156 ± .021	16.9 ± 6.06	16.8 ± 5.93	16.9 ± 6.05
	LR	0.314 ± .002	0.148 ± .003	11.7 ± .857	11.8 ± .796	11.7 ± .846
	LR-recurrence	0.281 ± .003	0.126 ± .008	10.0 ± 1.55	10.0 ± 1.55	10.0 ± 1.55
	LR-HDPS	0.324 ± .009	0.152 ± .016	12.8 ± 2.55	12.8 ± 2.55	12.8 ± 2.55

PS denotes the MAE of the propensity score. PS_W_ represents the weighted MAE of the propensity score. ATE_T_ and ATE_C_ denote ATE estimates with symmetric trimming and clipping, respectively. We report the mean values with their 95% confidence intervals, computed using 10-fold cross-validation. MLP and LR indicate logistic regression and multilayer perceptrons, respectively. MLP-recurrence and LR-recurrence denote logistic regression and multilayer perceptrons with additional recurrence features. MLP-HDPS and LR-HDPS denote logistic regression and multilayer perceptrons with high-dimensional propensity score adjustment. Bold values indicate the best performance across all models and baselines.

**Table 2.** Mean absolute error (MAE) of propensity scores and average treatment effect (ATE) estimates across different methods and baselines on the semi-synthetic dataset.

Model	PS	PS_W_	ATE	ATE_T_	ATE_C_
LSTM	**0.112** *±.*021	**0.062** *±.*014	0.677 *±* 1.15	0.659 *±* 1.10	**0.496 ** *±* ** **1.00
BERT_code_	0.156*±.*016	0.078*±.*012	0.782 *±* 1.27	0.769 *±* 1.25	0.781 *±* 1.26
BERT_record_	0.156*±.*009	0.077*±.*004	**0.597 ** *±* ** **1.05	**0.575 ** *±* ** **1.00	0.550 *±* 1.02
MLP	0.171*±.*002	0.094*±.*004	1.26*±.*671	1.27*±.*687	1.26*±.*702
MLP-recurrence	0.171*±.*003	0.094*±.*003	1.11*±.*448	1.07*±.*327	1.07*±.*332
MLP-HDPS	0.210±.009	0.114±.009	1.95 ± 1.17	1.93 ± 1.14	1.96 ± 1.15
LR	0.172*±.*003	0.094*±.*004	1.27 *±* 1.54	1.09*±.*786	1.09*±.*988
LR-recurrence	0.170*±.*002	0.094*±.*005	1.17*±.*989	1.16*±.*978	1.18*±.*966
LR-HDPS	0.212±.011	0.114±.009	1.94 ± 1.27	1.92 ± 1.26	1.92 ± 1.29

Notations are consistent with Table 1. We report the mean values with their 95% confidence intervals, computed using 10-fold cross-validation. Bold values indicate the best performance across all models and baselines

The attention weights of the Transformer encoder and decoder can provide explanations of the model.^17^^,^^43^ Therefore, we calculate the average attention weights assigned to confounding variables, all other variables, and [CLS] token to assess whether the attention weights in BERT_record_ and BERT_code_ can offer useful information for identifying confounding variables. Since we utilize the final representation corresponding to the [CLS] token to estimate the propensity score, the attention weights from the [CLS] token at the last encoder layer are used to calculate the average attention weights. The results are presented in Table 3. We observe that the confounding variables receive significantly higher attention weights compared to all other variables and [CLS] token, providing interpretability to the results using BERT_record_ and BERT_code_.

**Table 3.** The average attention weights assigned to confounding variables, all other variables, and [CLS] token.

Dataset	Confounding variables		All other variables		[CLS] token	
	BERT_code_	BERT_record_	BERT_code_	BERT_record_	BERT_code_	BERT_record_
Synthetic-CO	**0.130**±.014	**0.118**±.009	0.060±.005	0.011±.001	0.066±.015	0.042±.015
Synthetic-OD	**0.276**±.068	**0.200**±.036	0.013±.002	0.062±.005	0.022±.006	0.055±.012
Synthetic-OW	**0.156**±.037	**0.171**±.040	0.009±.002	0.069±.007	0.028±.007	0.061±.018
Semi-synthetic	**0.186**±.034	**0.215**±.060	0.091±.016	0.114±.030	0.208±.025	0.123±.037

We report the average values with 95% confidence intervals calculated using 10-fold cross-validation on the synthetic dataset and 5-fold cross-validation on the semi-synthetic dataset. Synthetic-CO, Synthetic-OD, and Synthetic-OW denote synthetic dataset with *consecutive occurrence*, *occurrence distance*, and *occurrence window* scenario, respectively. Bold values indicate the highest attention weights for each dataset and model.

## Discussion

Based on the results of 2 sets of experiments, we demonstrate that deep sequence models (LSTM and BERT) consistently outperform the baselines. This highlights the ability of deep sequence models to capture temporal patterns of confounders, leading to more precise ATE estimation. While LSTM generally performs better than BERT, we believe this is due to the relatively simple temporal patterns of the confounding variables and the limited complexity of the data (eg, shorter record lengths and fewer codes). We conjecture that BERT could achieve better performance with more complex data and temporal patterns of confounders. This is supported by previous studies that have demonstrated the superiority of BERT over RNNs in various medical tasks using EHRs.^34–37^

While BERT_code_ generally shows better performance compared to BERT_record_, we also believe that BERT_code_ may encounter challenges when dealing with claims records that have longer histories. Since BERT is known to struggle with learning from long sequences,^32^^,^^44^ BERT_code_ might face difficulties in learning from records observed over long periods of time, as the input sequence length of BERT_code_ increases faster than that of BERT_record_. We do not observe this issue for the BERT_code_ in our experiments, possibly because our datasets were not particularly long, with an average number of total codes per sample being less than 40 (see the Supplementary Material). However, conducting experiments using claims records containing longer histories, as well as devising efficient representations of a record beyond simple average pooling, remains a subject for future research.

We find that using HDPS for feature processing does not significantly improve the performance of the baseline methods. Our results show that HDPS only slightly improves the performance of logistic regression, as shown in Tables 1 and 2. This underscores that the temporal pattern of the confounders cannot be captured readily by rule-based feature processing. Moreover, we observe that extreme values of estimated propensity scores do not occur frequently enough to have a significant impact on the estimation of ATE, as evidenced by the similar values of MAE of ATE with and without symmetric trimming and clipping.

**Figure 3.**
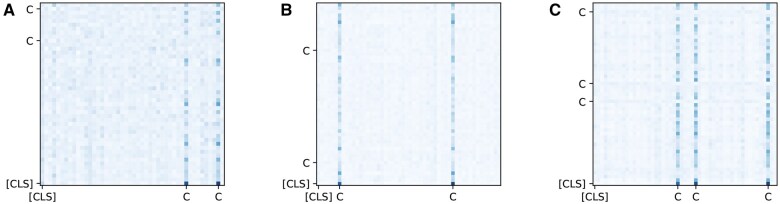
Visual representation of attention weights at the last encoder layer of ${\text{BERT}}_{\text{code}}$ for selected samples from the test set. C indicates the position of confounding variables. [CLS] indicates the position of [CLS] token. Darker color represents higher attention weight. (A). Synthetic dataset-CO. (B). Synthetic dataset-OD. (C). Synthetic dataset-OW.

We demonstrate the interpretability of BERT by showing that the confounding variables receive significantly higher attention weights than all other variables. Figure 3 provides a visual representation of attention weights at the last encoder layer of BERT_code_ for selected samples from the test set in each experiment. We observe that [CLS] token pays more attention to the positions of confounding variables. This highlights the utility of BERT in identifying potential confounders in observational data, particularly in cases where prior information about confounders is limited.

## Conclusion

In this study, we empirically demonstrate the effectiveness of deep learning models in estimating treatment effect on claims records using inverse probability of treatment weighting. Unlike existing methods that require feature processing for propensity score estimation, our study finds that deep sequence models can achieve better performance in estimating propensity score without the need for feature processing. Furthermore, we find that the interpretability of BERT can be used to identify potential confounders even when prior information about confounders is limited, offering practical utility. Based on these findings and results, we believe that inverse probability of treatment weighting using deep sequence models presents a promising approach for treatment effect estimation within the context of our problem setup.

Our study has a few limitations. First, the problem setup for treatment effect estimation is more complex in practice than in our setup. For instance, multiple treatments are often administered rather than a single binary treatment in real-world situations. Moreover, estimating treatment effect over multiple time points would be more practical and useful. Second, our synthetic and semi-synthetic datasets do not fully replicate the complexity of real-world claims records. While we carefully designed multi-step processes with considerations of static and dynamic variables in generating the synthetic dataset, real-world claims records involve countless factors such as disease-disease and drug-disease interactions. Furthermore, our semi-synthetic dataset only considers one hypothetical scenario and may not be a representative example of real-world data.

In future works, it is important to address the limitations identified in this study. This includes developing deep sequence models that can accurately estimate treatment effect in more complex scenarios, such as when multiple treatments are involved or when estimation is needed over multiple time points. Additionally, applying propensity scores estimated by deep sequence learning models to other methods, such as propensity score matching, will be another promising direction for future research. Inspired by recent efforts to model irregular time-series data using neural ordinary differential equations,^45^^,^^46^ modeling claims records in a similar way could be a valuable approach. Furthermore, applying our methods to real-world claims records will offer valuable insights into the clinical relevance and applicability of our findings.

## Supplementary Material

ooaf032_Supplementary_Data

## Data Availability

The code and data are available at *github.com/Jayaos/propensity_score_dl*.
