# Supplementary material for: Accurate treatment effect estimation using inverse probability of treatment weighting with deep learning
Source: JAMIA Open. 2025 Apr 26;8(2):ooaf032. doi: 10.1093/jamiaopen/ooaf032 (PMC12033031; doi:10.1093/jamiaopen/ooaf032)
Supplement: ooaf032_Supplementary_Data [file ooaf032_supplementary_data.zip › DLPS_jamia_open_supplementary_rev1_nomarkup.docx]

**ADDITIONAL DATASET DETAILS**

**Supplementary Table 1.** Summary statistics of the synthetic and semi-synthetic dataset.

| **Dataset** | **Size** | **# of unique codes** | **Avg. record length** | **Avg. codes per sample** | **Avg. codes per record** | **Prev. treated** |
| --- | --- | --- | --- | --- | --- | --- |
| Synthetic-CO | 12000 | 100 | 11.64 | 40.69 | 3.46 | 0.54 |
| Synthetic-OD | 12000 | 100 | 10.10 | 32.00 | 3.18 | 0.47 |
| Synthetic-OW | 12000 | 100 | 9,99 | 37.05 | 3.60 | 0.48 |
| Semi-synthetic | 6864 | 127 | 5.55 | 5.76 | 1.03 | 0.52 |

**Synthetic Dataset**

The static variable for each dimension is generated from a uniform distribution on the interval $\left[ 5,10 \right]$, resulting in a static variable vector $b\in\left[ 5,10 \right]^{d_{x}}$. Since static variables do not change over time, $B$ is constructed as a matrix with $T$ identical rows of $b$. For the dynamic variables, we generate dynamic variable vector $c\in\left[ 240,260 \right]^{d_{x}}$, then construct a matrix $C$ with $T$ identical rows of $c$. To incorporate the time-varying property of dynamic variables, we use $\text{B-spline}\left( t \right)$ sampled from the mixture of five quartic splines to generate $T$-dimensional time-varying coefficient vector for $k$-th dimension as:

$$\left( \text{B-spline}_{k}\left( 1 \right),\text{B-spline}_{k}\left( 2 \right),\ldots,\text{B-spline}_{k}\left( T \right) \right),$$

and then multiply this to $k$-th column of $C$ (i.e., $C_{:,k}$). The five quartic splines represent mild incline, mild decline, mild decline after steep incline, mild incline after steep decline, and stable states, respectively. This sampling of B-spline to generate time-varying coefficients is motivated by Schulam and Saria,[1], and code for implementation can be found at github.com/Jayaos/propensity_score_dl.

Consecutive occurrence scenario

In the *consecutive occurrence* scenario, high propensity scores and outcomes were assigned to samples having consecutive occurrences of a specific code. The true propensity score under the *consecutive occurrence* scenario is assigned as follows:

|  | $\sigma\left( o \right)+\epsilon$ | if $o > 1$ |
| --- | --- | --- |
| $e\left( X \right)=$ | $0.3 + \epsilon$ | if $o = 1$ |
|  | $0.1 + \epsilon$ | if $o < 1$ |

where $o$ is the maximum number of consecutive occurrences of the specific code and $\sigma\left( \cdot\right)$ is a sigmoid function. The noise $\epsilon$ is i.i.d. generated from a normal distribution with zero mean and variance of 0.01. The untreated outcome is assigned as follows:

| $Y_{0}\left( X \right)=$ | $b + \alpha o + \epsilon$ | if $o > 1$ |
| --- | --- | --- |
|  | $b + \epsilon$ | if $o \leq1$ |

where $b$ is a base outcome and $\alpha$ is an outcome coefficient for the maximum number of consecutive occurrences. The noise $\epsilon$ is i.i.d. generated from a normal distribution with zero mean and variance of 0.1. We set $b=10$ and $\alpha=10$. The untreated outcome is assigned as follows:

| $Y_{0}\left( X \right)=$ | $b + \alpha o + \epsilon$ | if $o > 1$ |
| --- | --- | --- |
|  | $b + \epsilon$ | if $o \leq1$ |

Occurrence distance scenario

The *occurrence distance* scenario assigns high propensity scores and outcomes to samples with shorter record-wise distances between the occurrences of two specific codes. The true propensity score under the *occurrence distance* scenario is assigned as follows:

| $e\left( X \right)=$ | $\sigma\left( log\frac{10}{5d+1} \right)+\epsilon$ | if $o > 1$ |
| --- | --- | --- |
|  | $0.3 + \epsilon$ | if $o \leq1$ |

where $d$ is the shortest record-wise distance within the records. The noise $\epsilon$ is i.i.d. generated from a normal distribution with zero mean and variance of 0.01. When $d$ cannot be able to be obtained, in the case when one or both of the two specific codes did not occur within the records, we assign $0.3 + \epsilon$. The untreated outcome is assigned as follows:

| $Y_{0}\left( X \right)=$ | $b+\frac{\alpha}{d+1}+\epsilon$ | if $d \geq0$ |
| --- | --- | --- |
|  | $b + \epsilon$ | otherwise |

where $b$ is a base outcome and $\alpha$ is an outcome coefficient for the shortest occurrence distance. The noise $\epsilon$ is i.i.d. generated from a normal distribution with zero mean and variance of 0.1. We set $b=10$ and $\alpha=40$.

Occurrence window scenario

In the *occurrence window* scenario, samples with more occurrences within a specific lookup window are assigned with high propensity scores and outcomes. We set the lookup window as the last three records of samples. The true propensity score under the *occurrence window* scenario is assigned as follows:

| $e\left( X \right)=$ | $\sigma\left( c \right)+\epsilon$ | if $c > 1$ |
| --- | --- | --- |
|  | $0.1 + \epsilon$ | if $c = 0$ |

where $c$ is the number of occurrences of the code within the lookup window. The untreated outcome is generated as follows:

$$Y_{0}\left( X \right)=b+\alpha c+\epsilon$$

where $b$ is a base outcome and $\alpha$ is an outcome coefficient for the occurrence count of the code within the lookup window. The noise $\epsilon$ is i.i.d. generated from a normal distribution with zero mean and variance of 0.1. We set $b=10$ and $\alpha=10$.

Since the samples having meaningfully high confounding rarely occurs in the synthetic dataset, we randomly selected five codes to have increasing probability of occurrences then selected codes for generating confounding among the selected codes. In all scenarios, the treatment effect is identically set to -5, which results in $Y_{0}-Y_{1}=-5$ for all samples in any confounding scenario. **Supplementary Figure 1a-c** displays the distributions of the true propensity scores associated with the three confounding scenarios. **Supplementary Table 1** shows the summary statistics of the synthetic dataset with three confounding scenarios.

**Semi-synthetic Dataset**

With the semi-synthetic dataset, we consider a hypothetical scenario where we aim to estimate the treatment effect for *viral sinusitis* and modified Synthea to introduce time-dependent confounding as follows: we identified two disease codes, *chronic sinusitis* and *viral sinusitis*; and assigned higher true propensity scores and outcomes to samples with a shorter record-wise distance between occurrences of these two codes. This scenario is similar to the *occurrence distance* scenario of the synthetic dataset. The true propensity score is assigned as follows:

$$e\left( X \right)=\sigma\left( 2log\frac{10}{d^{2.5}} \right)+\epsilon,$$

where $d$ is the shortest record-wise distance between *chronic sinusitis* and *viral sinusitis* within the records. The noise $\epsilon$ is i.i.d. generated from a normal distribution with zero mean and variance of 0.01. Note that the case when $d=0$ (i.e., *chronic sinusitis* and *viral sinusitis* occur in the same record) was not observed in the dataset. The untreated outcome is generated as follows:

$Y_{0}\left( X \right)=b+\frac{\alpha}{d}+\epsilon,$

where $b$ is a base outcome and $\alpha$ is an outcome coefficient for the shortest occurrence distance. The noise $\epsilon$ is i.i.d. generated from a normal distribution with zero mean and variance of 0.1. We set $b=10$ and $\alpha=5$. **Supplementary Figure 1d** displays the distribution of the true propensity scores. **Supplementary Table 1** shows the summary statistics of the semi-synthetic dataset.

**Sample Data**

Supplementary Table 2 shows the two sample data in the semi-synthetic dataset. The patient with patient id ‘36d131ee-dd5b-4acb-acbe-19961c32c099’ has three visits (i.e., encounters), where the first visit ‘ff93cedb-8245-4091-838d-a568bcbcb00b’ contains *Viral sinusitis*, the second visit ‘ffdddbfb-35e8-4a74-a801-89e97feed2f3’ contains *Viral sinusitis*, and the last visit ‘4620bd2f-8010-46a9-82ab-8f25eb621c37’ contains *Acute viral pharyngitis*. The patient with patient id ‘6e9f8b3e-5a21-401e-868d-2d62e0e7f452’ has two visits, where the first visit ‘dd8f1a5a-9f6c-4418-8b88-c49c49377b6c’ contains *Chronic sinusitis*, *Normal pregnancy*, *Miscarriage in second trimester*, *Fetus with chromosomal abnormality* and the second visit ‘beb31ddd-3f24-4e9d-a8fc-ade120227dbc’ contains *Viral sinusitis*.

If we assume that there are only six unique medical codes (*Viral sinusitis*, *Acute viral*, *Chronic sinusitis*, *Normal pregnancy*, *Miscarriage in second trimester*, and *Fetus with chromosomal abnormality pharyngitis*), each visit of a patient can be represented as a 6-dimensional multi-hot vector, where each dimension corresponds to a unique medical code. For example, the first and second visits of the patient with patient id ‘36d131ee-dd5b-4acb-acbe-19961c32c099’ is represented as [1,0,0,0,0,0], where the first element indicates the presence of *Viral sinusitis*. The first visit of the patient with patient id ‘6e9f8b3e-5a21-401e-868d-2d62e0e7f452’ is represented as [0,0,1,1,1,1], where the third to sixth elements indicate the presence of *Chronic sinusitis*, *Normal pregnancy*, *Miscarriage in second trimester*, and *Fetus with chromosomal abnormality*, respectively.

**Supplementary Table 2.** Two randomly selected sample patients and their records from the semi-synthetic dataset.

| **Patient ID** | **Encounter ID** | **Encounter Date** | **Code** |
| --- | --- | --- | --- |
| 36d131ee-dd5b-4acb-acbe-19961c32c099 | ff93cedb-8245-4091-838d-a568bcbcb00b | 2011-05-07 | Viral sinusitis |
| 36d131ee-dd5b-4acb-acbe-19961c32c099 | ffdddbfb-35e8-4a74-a801-89e97feed2f3 | 2014-08-03 | Viral sinusitis |
| 36d131ee-dd5b-4acb-acbe-19961c32c099 | 4620bd2f-8010-46a9-82ab-8f25eb621c37 | 2016-10-04 | Acute viral pharyngitis |
| 6e9f8b3e-5a21-401e-868d-2d62e0e7f452 | dd8f1a5a-9f6c-4418-8b88-c49c49377b6c | 2010-02-20 | Chronic sinusitis,  Normal pregnancy,  Miscarriage in second trimester,  Fetus with chromosomal abnormality |
| 6e9f8b3e-5a21-401e-868d-2d62e0e7f452 | beb31ddd-3f24-4e9d-a8fc-ade120227dbc | 2011-04-20 | Viral sinusitis |

**ADDITIONAL IMPLEMENTATION DETAILS**

**Hyperparameters**

We determined the optimal hyperparameters for all models and baselines by using validation and training sets. **Supplementary Table 3** shows a list of hyperparameters and their optimal values for all models and baselines. For BERT, the feed-forward dimension was set to 4 times the model dimension. We did not use dropout for LSTM, BERT, and MLP.

**Supplementary Table 3.** List of hyperparameters and their optimal values for all models and baselines.

| **Model** | **Hyperparameters and their optimal values** |
| --- | --- |
| LSTM | Embedding dimension = 64  Hidden dimension = 64  Number of LSTM layers = 2  Learning rate = 0.00001  Batch size = 16 |
| BERT_code_ | Embedding dimension = 64  Model dimension = 64  Number of encoder layers = 2  Number of attention heads = 4  Learning rate = 0.00001  Batch size = 16 |
| BERT_record_ | Embedding dimension = 64  Model dimension = 64  Number of encoder layers = 2  Number of attention heads = 4  Learning rate = 0.00001  Batch size = 16 |
| MLP | Embedding dimension = 64  Number of hidden layers = 2  Hidden units = 64  Learning rate = 0.0001  Batch size = 16 |
| MLP-recurrence | Embedding dimension = 64  Number of hidden layers = 2  Hidden units = 64  Learning rate = 0.0001  Batch size = 16 |
| MLP-HDPS | Embedding dimension = 64  Number of hidden layers = 2  Hidden units = 64  Learning rate = 0.00001  Batch size = 16 |
| LR | Learning rate = 0.001  Batch size = 16 |
| LR-recurrence | Learning rate = 0.001  Batch size = 16 |
| LR-HDPS | Learning rate = 0.0001  Batch size = 16 |

**High-dimensional Propensity Score Adjustment for Baselines Methods**

High-dimensional Propensity Score Adjustment (HDPS) is a multi-step algorithm used to implement high-dimensional proxy adjustment in claims records,[2]. HDPS first identifies candidate features based on the feature prevalence and then generates recurrence features. The recurrence features are three new binary variables that capture the recurrence patterns of each feature within a sample’s records: (1) whether the code occurred in the records at least once; (2) whether the code occurred in the records more than the median number of occurrences; and (3) whether the code occurred in the records more than the 75th percentile number of occurrences. For each of these three covariates, a value of 1 indicates "True" and 0 indicates "False." The median and 75th percentile values are computed from the entire dataset. This recurrence feature generation increases the total number of features to three times the number of unique codes in the dataset. Next, HDPS ranks and selects the top k features with the highest potential to control confounding, measured by computing bias for each feature, which is defined as:

$$bias=\frac{P_{C1}\left( \alpha-1 \right)+1}{P_{C0}\left( \alpha-1 \right)+1}\quad\text{if}\quad\alpha\geq1,$$

$$bias=\frac{P_{C1}\left( \frac{1}{\alpha}-1 \right)+1}{P_{C0}\left( \frac{1}{\alpha}-1 \right)+1}\quad\text{if}\quad\alpha<1,$$

where $P_{C1}$ and $P_{C0}$ represent the prevalence of the feature among the treated and untreated samples, respectively. $\alpha$ denotes the independent association between the feature and the outcome. We use linear regression to compute $\alpha$ for all features.

In our study, we apply HDPS as a feature processing method for the baseline models (i.e., logistic regression and MLP). We conduct experiments using both the original HDPS setup, as described in the original publication, and an implementation of HDPS without feature selection [2].

**REFERENCES**

[1] Peter Schulam and Suchi Saria. Reliable decision support using counterfactual models. Advances in neural information processing systems, 30, 2017.

[2] Sebastian Schneeweiss, Jeremy A Rassen, Robert J Glynn, Jerry Avorn, Helen Mogun, and M Alan Brookhart. High-dimensional propensity score adjustment in studies of treatment effects using health care claims data. Epidemiology, 20(4):512–522, 2009.

**Supplementary Figure 1.** Propensity score distributions for the synthetic dataset under the (a) consecutive occurrence, (b) occurrence distance, and (c) occurrence window scenarios, as well as (d) the semi-synthetic dataset. Gray bars represent treated samples, while unshaded bars denote untreated samples.
