# Supplementary figures and images for: Accurate treatment effect estimation using inverse probability of treatment weighting with deep learning
Source: JAMIA Open. 2025 Apr 26;8(2):ooaf032. doi: 10.1093/jamiaopen/ooaf032 (PMC12033031; doi:10.1093/jamiaopen/ooaf032)

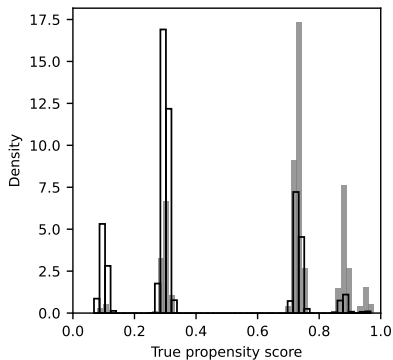

(a)

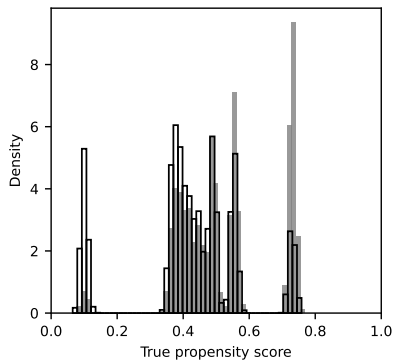

(b)

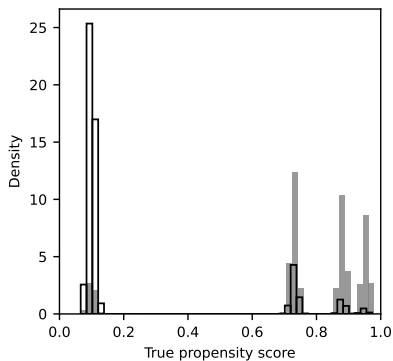

(c)

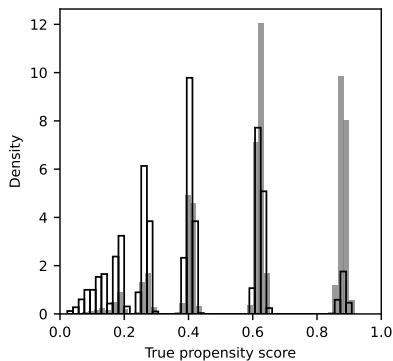

(d)

Supplement: ooaf032_Supplementary_Data [file ooaf032_supplementary_data.zip › DLPS_supfigure1.pdf]
